# A comprehensive analysis of brain network complexity in task-based fMRI using entropy: systematic review

**DOI:** 10.1007/s11682-026-01124-y

**Published:** 2026-03-26

**Authors:** Jeonghoon Park, Nan Xu, Maysam Nezafati

**Affiliations:** 1 Georgia Institute of Technology, 313 Ferst Dr NW, GA 30332 Atlanta, USA; 2 University of Maryland, College Park, MD USA

**Keywords:** Functional magnetic resonance imaging (fMRI), Brain entropy, Neural complexity, Shannon entropy, Sample entropy, Multiscale entropy, Cognitive function

## Abstract

Entropy-based analysis is increasingly used in task-based functional magnetic resonance imaging (fMRI) to quantify neural signal complexity and information dynamics, but variation in entropy definitions, parameter choices, and analytic scope can limit cross-study comparability. To systematically review how entropy measures are implemented, parameterized, and interpreted in task-based fMRI studies in healthy human subjects, focusing on methodological practice. Web of Science was searched using the keywords “fMRI” and “entropy” for the period 2000–2023, restricted to journal articles, proceedings papers, review articles, meeting abstracts, and book chapters. Included studies used task-based fMRI, applied entropy-based quantitative measures, involved healthy human participants, and reported original empirical findings or methodological applications. Non-human, clinical, and resting-state studies were excluded. Records were screened by verifying whether “fMRI” and “entropy” appeared in the title, keywords, Keywords Plus, or abstract. Extracted items included entropy type, analytic scope (regional/voxel-wise, network-level, connectivity-based), parameter and reporting details, task types, and preprocessing context where available. Data were synthesized using structured narrative methods because meta-analysis was not appropriate given differences in entropy definitions, parameterization, task types, and outcome metrics. Risk of bias was assessed with an adapted Joanna Briggs Institute (JBI) checklist (Joanna Briggs Institute, 2017). Database searches yielded 1,313 records. 274 were screened and 234 full texts assessed. 92 studies met inclusion criteria. Exclusions at full-text were primarily resting-state studies (*n* = 81), clinical populations (*n* = 42), and non-human studies (*n* = 19). Across the 92 included studies, Shannon entropy predominated (78.3%), followed by sample entropy (9.78%), transfer entropy (4.35%), multiscale entropy (3.26%), approximate entropy (3.26%), and multiple-entropy approaches (1.09%). Entropy measures were found to be matched with distinct methodological roles. Approximate and sample entropy were commonly used for regional or voxel-wise signal regularity, multiscale entropy for multi–time scale complexity (often at the network level), transfer entropy for directed connectivity, and Shannon entropy for broad applications including machine-learning feature and validation use. Evidence synthesis was constrained by inconsistency in entropy formulations, parameter reporting, preprocessing decisions, and outcome metrics. Formal heterogeneity testing, subgroup analyses, and sensitivity analyses were not conducted, and results were summarized descriptively. Task-based fMRI entropy research is methodologically diverse but consistently demonstrates the feasibility of using entropy to characterize task-related brain complexity across different analytic levels. The prevalent use of Shannon entropy and inconsistent parameter/reporting practices underscore the need for clearer, standardized reporting and reproducible implementation guidance to improve comparability across studies.

## Introduction

Brain imaging plays an important role in evaluating the neurological state of an individual (Brody et al., 2015). One of the commonly used neuroimaging tools is functional magnetic resonance imaging (fMRI), which uses Blood Oxygen Level Dependent (BOLD) signals to quantify the changes in neural state (Biswal et al., 1995). With the advent of fMRI, various ways to better analyze brain dynamics have been developed, including entropy-based analysis (Keilholz et al., 2020; Keilholz et al., 2017; Nezafati et al., 2020).

Brain entropy is defined as the randomness of brain signals. Due to its capability of capturing brain networks and their dynamics effectively (Fagerholm et al., 2023), analysis based on entropy is often regarded as a promising method to quantify the brain’s complex dynamics and functions (Samantaray et al., 2025). This method is especially useful when investigating task-based cognitive neural functions (Najafi et al., 2016) as they are often intertwined with brain complexity and variability (Ji et al., 2022; Keshmiri, 2020), ranging from emotional face-matching tasks to simple finger-tapping tasks (Savage et al., 2024).

Using fMRI imaging and entropy-based analyses together allows researchers to map patterns within information-rich fMRI data and investigate how different types of cognitions are associated with specific brain regions (Deco et al., 2021; Song et al., 2019). By capturing the temporal dynamics and spatial connectivity of fMRI data, this analysis tool can provide additional insights on top of functional connectivity and activation mapping (Doganci et al., 2023; Hlinka et al., 2011).

The goal of this study is to systematically and comprehensively review the application of various types of entropy measures to fMRI brain signals, in order to identify whether this analytical tool is reliable and effective in enhancing our understanding of brain variability and functions under task-based conditions. It examines how different entropy formulations are implemented across cognitive task conditions, including their parameter choices, analytic scope (regional, network-level, or connectivity-based), and interpretations. By synthesizing current practices and highlighting the methodological inconsistencies that limit cross-study comparability, this review seeks to provide practical guidance for the standardized and reproducible use of entropy measures in future fMRI studies.

## Methods

**Fig. 1:**
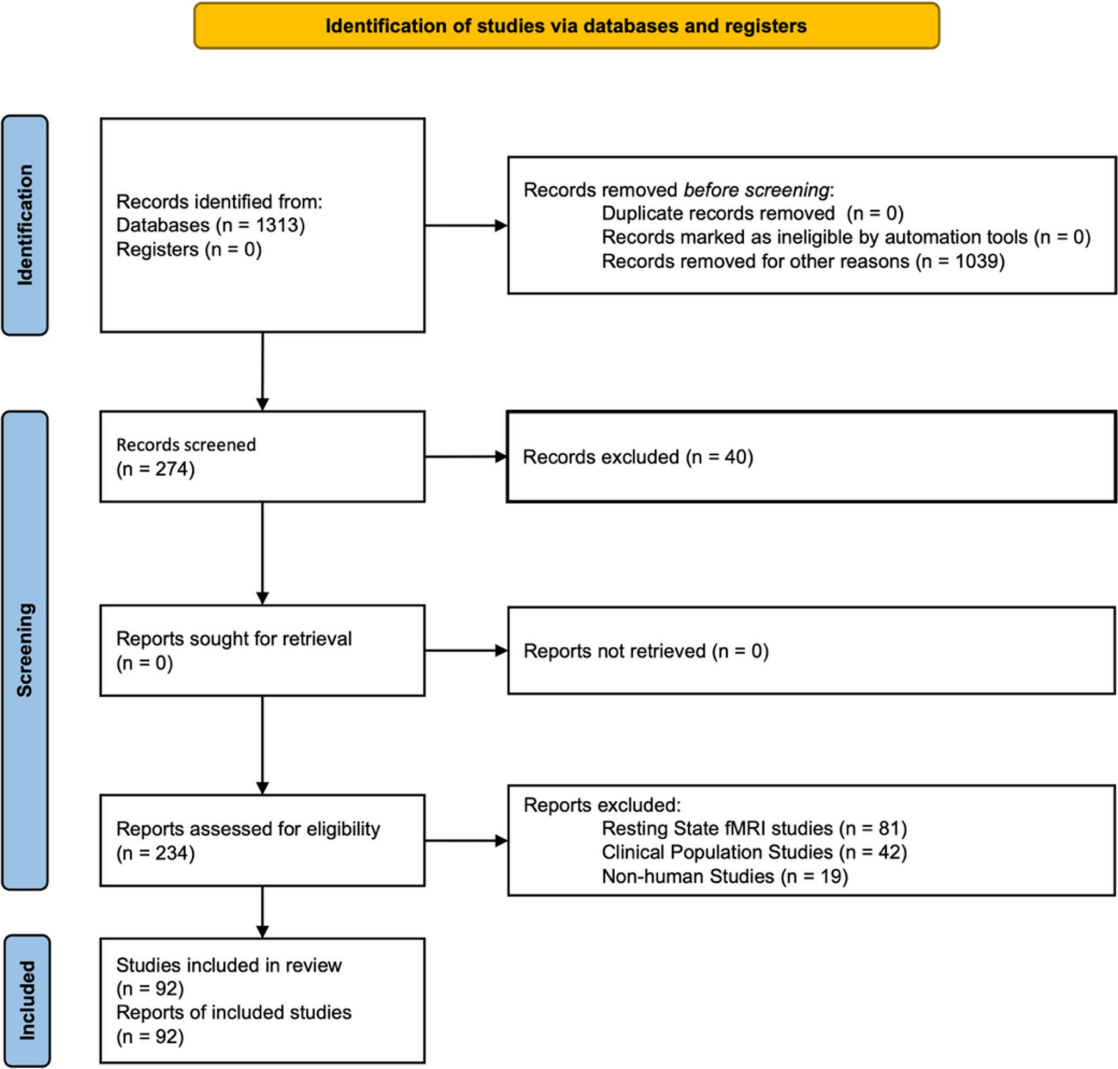
Visual representation of analysis process

This study was conducted as a systematic methodological review of entropy-based analyses applied to task-based functional magnetic resonance imaging (fMRI). The review protocol and reporting follow the Preferred Reporting Items for Systematic reviews and Meta-Analyses (PRISMA) 2020 guidelines for systematic reviews (Page et al., 2021; Takkouche & Norman, 2011). A PRISMA flow diagram summarizing the identification, screening, eligibility, and inclusion of studies is provided in Fig. 1. Importantly, this review was designed to evaluate how entropy measures are implemented, parameterized, and interpreted in task-based fMRI.

The relevant scientific articles were searched using “fMRI” and “entropy” as keywords from the Web of Science database as the main source within the timeframe from year 2000 to 2023. The search was also limited to journal articles, proceeding papers, review articles, meeting abstracts, and book chapters. Metadata of these papers were exported to an Excel file and then filtered using four criteria, predefined to align with the aim of the study. Studies were included if they met all of the following criteria:

1. Used fMRI data.
2. Applied entropy-based quantitative measures.
3. Used task-based experiments.
4. Only included healthy human subjects.
5. Reported original empirical findings or methodological applications.

Although non-human and resting-state studies were initially identified during screening, they were excluded from the final analysis and are reported only in the screening summary for transparency.

These papers were screened by verifying whether the keywords “fMRI” and “entropy” appeared in any of the following: (1) the title, (2) the authors’ keywords, (3) the “Keywords Plus” field, or (4) the abstract. Studies that did not meet any of these criteria were excluded (see Fig. 1).

For each included study, the following methodological information was extracted:

1. Entropy measures used.
2. Analytical scope (regional, network-level, or connectivity-based).
3. Details on parameters.
4. Type of tasks.
5. fMRI preprocessing and analysis context if available.
6. Study objective related to applying entropy.

Data extraction focused on methodological implementation rather than cognitive interpretation, in line with the technical focus of this review.

Based on this information, studies were classified based on three main categories: (1) subject type (healthy vs. clinical populations), (2) cognitive domain (resting-state and the six DSM-5-defined domains), and (3) entropy type (see Table 1).

**Table 1:** Article categorization

Modality	Subject	Cognitive state	Entropy type
Entropy Key Papers	Healthy human subjects	Resting-State	Approximate Entropy
fMRI	Clinical Population	Complex Attention	Sample Entropy
EEG		Executive Function	Multiscale Entropy
fMRI & EEG		Learning and Memory	Transfer Entropy
Out of scope		Language	Shannon Entropy
		Perceptual-motor Control	Multiple Entropy methods
		Social Cognition	
		Multiple Cognitive Tasks	

For this systematic literature review, studies that have used fMRI on healthy human subjects under non-resting-state conditions were selected. These papers were then analyzed both quantitatively and qualitatively, focusing on how entropy measures are applied to the research. For further qualitative analysis, to illustrate representative implementations, highly cited studies were used as examples of methodological practice.

Following screening and eligibility assessment, 92 studies met all inclusion criteria and were included in the final review. These studies form the basis of the methodological synthesis presented in the Results section. Data were synthesized using a structured narrative approach, as meta-analysis was not appropriate due to discrepancies in entropy definitions, parameterization, task types, and outcome metrics. No data transformations or imputations were performed. Results were presented using summary tables grouped by entropy type and analytic level, alongside a PRISMA flow diagram. Methodological gaps were explored qualitatively by comparing entropy formulations, parameter reporting practices, and analytic scope. Formal statistical heterogeneity testing, subgroup analyses, and sensitivity analyses were not conducted.

Risk of bias was assessed at the study level using an adapted Joanna Briggs Institute (JBI) Critical Appraisal Checklist for Analytical Cross-Sectional Studies (Joanna Briggs Institute, 2017), selected due to the observational, task-based nature of the included fMRI studies. The tool was adapted to include entropy-specific reporting items. The following domains were evaluated: participant selection, task design clarity, fMRI acquisition and preprocessing transparency, entropy method specification, statistical analysis, confounding control, and outcome reporting. Each domain was rated as low, unclear, or high risk of bias. An overall qualitative risk-of-bias judgment (low, moderate, high) was assigned based on the number and severity of high-risk domains. Two reviewers independently assessed all studies, with disagreements resolved by consensus. No automation tools were used, and study authors were not contacted. Moreover, no standardized effect measures were synthesized. Included studies reported mixed entropy-based outcomes, such as entropy values, correlations, classification metrics, derived from non-comparable formulations. Results were therefore summarized descriptively without defining thresholds for effect magnitude. Furthermore, risk of bias due to missing results was assessed qualitatively by comparing stated objectives with reported outcomes and noting selective reporting of entropy parameters or results. Formal statistical methods were not applied due to the absence of quantitative effect estimates.

Certainty in the body of evidence was assessed narratively, as formal tools such as GRADE are not well suited to methodological reviews without intervention effects. Certainty judgements considered consistency of methodological findings, transparency of entropy implementation, reproducibility across studies, and overall risk of bias. The results of this assessment are summarized in a Summary of Findings table, which reports entropy type, analytic focus, consistency of methodological application, and overall confidence in the evidence. Certainty levels were therefore interpreted qualitatively and used to contextualize methodological conclusions.

## Entropy

Entropy measures unpredictability in a system and have been important emerging tools in quantifying brain complexity, specifically in fMRI research. There are various entropy types, each with unique characteristics and applicability (see Table 2). Below, we summarize key entropy measures, their principles, and their differences.

### Approximate entropy (ApEn)

Approximate Entropy (ApEn) measures how predictable a time series is by examining the persistence of similarity in sequences of data points over time. Lower ApEn values reflect greater regularity (such as highly predictable neural signals), while higher values indicate increased complexity (Delgado-Bonal & Marshak, 2019). Despite its usefulness, ApEn has important drawbacks, including dependence on parameter selection and biases from self-matching.

### Sample entropy (SampEn)

Sample Entropy (SampEn) improves upon Approximate Entropy (ApEn) by removing the issue of self-matching, which results in more reliable estimates across different datasets (Olbrys & Majewska, 2022). While both methods assess the regularity of time series, SampEn is less sensitive to data length and parameter choices, making it especially suitable for fMRI studies (Jia et al., 2017). In particular, it provides more stable outcomes for shorter time series compared to ApEn (Molina-Picó et al., 2011).

### Multiscale entropy (MSE)

Multiscale Entropy builds on Sample Entropy by assessing signal complexity across several time scales instead of just one (Costa et al., 2005). This method enables researchers to examine both short- and long-range patterns of neural variability, offering valuable insight into hierarchical brain dynamics. In contrast to ApEn and SampEn, which are limited to a single scale, MSE delivers a more comprehensive perspective by capturing entropy at multiple temporal resolutions (Gale et al., 2021).

### Shannon entropy (SE)

Shannon Entropy, derived from information theory, quantifies the uncertainty within a dataset by measuring the amount of information a signal carries (Shannon, 1948). Unlike ApEn, SampEn, or MSE, which evaluate temporal patterns and predictability in time-series data, Shannon entropy captures the overall randomness of neural activity without accounting for temporal structure (De Araujo et al., 2003). Because of this characteristic, advanced statistical methods - such as variational Bayesian inference - have increasingly been applied in classification and machine learning studies that use Shannon Entropy (Brodersen et al., 2013; Du & Fan, 2013).

### Transfer entropy (TE)

Transfer Entropy measures how information flows directionally from one time-series signal to another (Friston et al., 2013). This makes it especially valuable for evaluating functional connectivity in the brain, as it can identify how activity in one neural region drives changes in another over time (Ursino et al., 2020). Unlike other entropy-based methods, TE specifically captures causality and directional information transfer, providing a reliable framework for studying brain network dynamics (Frässle et al., 2017; Santos et al., 2017).

**Table 2:** Mathematical equations of Approximate Entropy, Sample Entropy, Multiscale Entropy, Transfer Entropy, and Shannon Entropy

Approximate Entropy (Pincus, 1991)	$ApEn\left(m,r\right)$=$\underset{n\to{\infty}}{\mathrm{lim}}\left[{\varphi}^{m}\left(r\right)-{\varphi}^{m+1}\left(r\right)\right]{\varphi}^{m}\left(r\right)$=$\frac{1}{N-m+1}{\sum}_{i=1}^{N-m+1}\mathrm{log}{C}_{i}^{m}\left(r\right)$ ${C}_{i}^{m}\left(r\right)$: probability of one vector to be close to another vector with the same length $m$: length of the template,$r$: de facto noise filter
Sample Entropy (Richman et al., 2004)	$SampEn$=$-\mathrm{log}\left(\frac{{\Sigma}{A}_{i}}{{\Sigma}{B}_{i}}\right)$ ${A}_{i}$: the number of template matches with vector${x}_{m+1}$ ${B}_{i}$= the number of template matches with${x}_{m}$
Multiscale Entropy (Costa et al., 2005; Humeau-Heurtier, 2015)	${y}_{j}^{\left(\tau\right)}$=$\frac{1}{\tau}{\sum}_{i=\left(j-1\right)\tau+1}^{j\tau}{x}_{i},1\le{j}\le\frac{N}{\tau}$ $MSE\left(x,\tau,m,r\right)$=$SampEn\left({y}_{j}^{\left(\tau\right)},m,r\right)$ $\tau$: scaling factor,${y}_{j}^{\left(\tau\right)}$: coarse-grained time series $m$: length of the template,$r$: de facto noise filter
Transfer Entropy (Schreiber, 2000)	$TE\left(X\to{Y}\right)$=$S\left(\frac{y\left(t\right)}{{Y}^{n}\left(t-{\Delta}t\right)}\right)$-$S\left(\frac{y\left(t\right)}{{X}^{m}\left(t-{\Delta}t\right)},{Y}^{n}\left(t-{\Delta}t\right)\right)$ $X,Y$: Time dependent vectors,$m,n$: embedding dimensions
Shannon Entropy (Shannon, 1948)	$H\left(X\right)=-{\sum}_{i=1}^{n}p\left({x}_{i}\right)\mathrm{log}\left(p\left({x}_{i}\right)\right)$ $p\left({x}_{i}\right)$: Probability of occurrence of${x}_{i}$

## Results

### Study selection

The study selection process followed the PRISMA 2020 guidelines and is summarized in the PRISMA flow diagram (Fig. 1). Database searches identified a total of 1,313 records. After removal of records that were deemed irrelevant for reasons unrelated to eligibility criteria (*n* = 1,039), 274 records remained and were screened based on titles and abstracts. No duplicate records were identified, and no records were excluded by automation tools. During the screening stage, 40 records were excluded based on title and abstract review. The remaining 234 reports were assessed for eligibility through full-text evaluation. Of these, 142 reports were excluded for the following predefined reasons: studies employing resting-state fMRI (*n* = 81), studies involving clinical populations (*n* = 42), and studies conducted in non-human subjects (*n* = 19). No reports were excluded due to retrieval issues. Following the eligibility assessment, 92 studies met all inclusion criteria and were included in the final systematic review. All included studies applied entropy-based analytical methods to task-based fMRI data collected from healthy human participants and reported original empirical findings or methodological applications relevant to entropy analysis. These 92 studies form the basis of the qualitative and quantitative synthesis presented in the subsequent sections of the Results.

### Study characteristics

The selected studies examined the application of entropy-based analytical methods to task-based fMRI data acquired from healthy human participants. All studies reported original empirical findings or methodological applications involving entropy measures and met the predefined inclusion criteria. The included studies spanned a wide publication range between 2000 and 2023, reflecting the progressive adoption of entropy-based techniques in task-based fMRI research. All studies employed experimental conditions designed to elicit task-related neural activity, with task structures varying in duration, complexity, and cognitive demands. No studies involved pharmacological manipulation, clinical interventions, or non-human subjects. Task conditions were diverse and covered a broad range of experimental contexts. For organizational purposes, studies were grouped according to task context into six DSM-5–defined cognitive domains (complex attention, executive function, learning and memory, language, perceptual–motor control, and social cognition), as well as a separate category for tasks involving multiple cognitive components. This categorization was used solely to summarize task contexts and does not imply theoretical distinctions or neural specificity. Perceptual–motor control tasks represented the largest proportion of included studies (See Table 3), followed by executive function and learning and memory tasks. Fewer studies investigated complex attention, language, and social cognition tasks. Several studies employed tasks that spanned multiple cognitive domains and were therefore classified accordingly.

**Table 3:** Quantitative literature review – categorization of healthy subjects based on cognitive domains

	Number of papers
Complex Attention	4
Executive Function	17
Learning & Memory	12
Language	6
Perceptual-motor Control	34
Social Cognition	6
Multiple	13
Total	92

A variety of entropy measures were applied across studies, with Shannon entropy being the most frequently used method, followed by sample entropy, transfer entropy, multiscale entropy, and approximate entropy. A subset of studies employed multiple entropy measures within the same analytical scope, most commonly combining Shannon entropy with sample entropy. Entropy analyses were conducted at different analytical levels, including regional or voxel-wise analyses, focusing on local signal regularity or variability, network-level analyses, assessing entropy across predefined functional networks or parcellations, and connectivity-based analyses, primarily using transfer entropy to estimate directed information flow between brain regions.

Considerable inconsistency was observed in reporting practices across studies. While most studies clearly specified the entropy measure used, the level of detail regarding parameter selection varied substantially. Similarly, descriptions of preprocessing steps and analytic pipelines ranged from comprehensive to minimal, limiting direct comparability across studies. Overall, the included studies represent a methodologically varying yet consistent body of literature under the same objectives which are to use entropy-based approaches to characterize task-related fMRI signals. These characteristics form the basis for the structured synthesis presented in the subsequent Results sections.

### Distribution of entropy measures used

Across the 92 included studies, a total of five primary entropy measures and their variations were identified, which were Shannon entropy, sample entropy, approximate entropy, multiscale entropy, and transfer entropy. In addition, a subset of studies employed combined entropy approaches, applying more than one entropy measure within the same analyses.

Shannon entropy and its extensions were the most frequently applied measures (78.3%), followed by sample entropy (9.78%), transfer entropy (4.35%), multiscale entropy (3.26%), approximate entropy (3.26%) and combined entropy approaches (1.09%)(See Table 4).This distribution indicates a strong methodological preference for Shannon entropy-based measures in task-based fMRI research, with other entropy measures applied more selectively depending on analytic goals.

**Table 4:** Quantitative literature review – entropy distribution of task-based fMRI studies

Entropy type	Number of Studies
Approximate Entropy	3
Sample Entropy	9
Multiscale Entropy	3
Shannon Entropy	72
Transfer Entropy	4
Multiple Entropy Types	1

Clear associations emerged between entropy type and analytic scope. Approximate entropy and sample entropy were predominantly applied in regional or voxel-wise analyses, focusing on local signal regularity and complexity (Wang et al. 2014b). Multiscale entropy was exclusively used to assess temporal complexity across multiple time scales, often within predefined brain networks (McDonough et al., 2019). Transfer entropy was consistently employed in connectivity-based analyses, examining directed information flow between regions or networks (Lizier et al., 2011). Shannon entropy demonstrated the broadest range of applications. It was used in regional, network-level, and whole-brain analyses, as well as in studies incorporating machine learning or classification, where entropy served either as a feature or as a validation metric (De Araujo et al., 2003; Richiardi et al., 2011).

Overall, the distribution of entropy measures across studies reflects distinct methodological roles rather than interchangeable use. Shannon entropy dominates the literature due to its conceptual simplicity and analytical flexibility, while sample entropy, multiscale entropy, and transfer entropy are applied in more specialized contexts. This uneven distribution underscores the importance of clearly defining entropy choice and analytic intent, a theme explored further in subsequent sections.

### Analytical scope of entropy applications

Across the included studies, entropy-based analyses were applied at multiple analytical scopes, reflecting different methodological objectives rather than differences in task content. These scopes can be broadly categorized into regional signal analyses, network-level analyses, and connectivity-based analyses. The choice of analytical scope was closely linked to the entropy measure employed and the specific research question addressed.

#### Regional and voxel-wise signal analyses

A substantial proportion of studies applied entropy measures at the regional or voxel-wise level, quantifying local signal regularity or variability within predefined regions of interest or across the whole brain. This approach was most commonly associated with approximate entropy and sample entropy, which are designed to assess the predictability and complexity of time-series signals. In these studies, entropy values were typically computed directly from BOLD time series extracted from individual voxels or anatomically or functionally defined regions (Duncan et al., 2015; Sokunbi et al., 2011). Analyses focused on comparing entropy values across task conditions, time periods, or participant groups. However, considerable variability was observed in how regions were defined, with studies employing voxel-wise analyses, atlas-based parcellations, or task-specific regions of interest. Parameter selection and reporting practices also varied widely, limiting direct methodological comparability across studies.

#### Network-level analyses

Network-level entropy analyses were used to characterize signal variability or complexity across large-scale functional networks rather than isolated regions. This analytical scope was most frequently associated with Shannon entropy and multiscale entropy. In these studies, entropy was computed after aggregating BOLD signals within predefined networks or parcellations, such as default mode, frontoparietal, or attention-related networks (Sen et al., 2019). Multiscale entropy analyses further extended this approach by examining entropy across multiple temporal resolutions, enabling the assessment of scale-dependent signal complexity (Gale et al., 2021; Omidvarnia et al., 2022). While network-level analyses allowed for broader characterization of task-related brain dynamics, substantial gaps were observed in network definitions, temporal scaling procedures, and aggregation strategies.

#### Connectivity and information-flow analyses

A smaller but distinct subset of studies applied entropy measures to assess functional interactions between brain regions, rather than local or network-averaged signals. These analyses primarily employed transfer entropy, which quantifies directed information flow between time series (Deco et al., 2021). Connectivity-based entropy analyses typically focused on estimating task-related changes in directional interactions between regions or networks. Transfer entropy was applied using both pairwise and multivariate structure, with embedding parameters and statistical testing procedures differing considerably across studies. Compared to regional and network-level approaches, connectivity-based analyses required more complex modeling choices and were more sensitive to parameter specification and data length constraints.

Overall, entropy-based fMRI studies tended to focus on different types of analyses depending on which entropy measure was used. Approximate and sample entropy were most often used to assess local signal regularity, multiscale entropy was applied to examine temporal complexity across scales, transfer entropy was reserved for directed connectivity analyses, and Shannon entropy was used flexibly across all three analytical levels. This diversity in analytical scope reflects the methodological versatility of entropy-based approaches but also contributes to substantial heterogeneity in implementation. These differences underscore the importance of clearly specifying analytic intent, scope, and parameterization when applying entropy measures in task-based fMRI research.

### Entropy applications across tasks conditions

For descriptive purposes, the included studies were grouped according to the task context in which entropy measures were applied. Task contexts were categorized using the six DSM-5 cognitive domains along with a separate category for tasks involving multiple cognitive components. This categorization was used solely as a organizational structure to summarize how entropy-based methods have been applied across different experimental settings and does not imply theoretical distinctions or domain-specific neural interpretations.

#### Complex attention

A small number of studies applied entropy measures in tasks involving sustained, divided, or selective attention. These studies predominantly employed Shannon entropy, typically at the regional or network level, to quantify task-related changes in signal variability (Yamashita et al., 2021). Due to the limited number of studies and methodological diversity, no consistent implementation pattern beyond the choice of entropy measure could be identified.

#### Executive function

Executive function tasks constituted a substantial portion of the included literature. Studies in this category employed a diverse set of entropy measures, including Shannon entropy, sample entropy, approximate entropy, and transfer entropy. Entropy analyses were applied at multiple analytical levels, ranging from voxel-wise signal regularity assessments to network-level complexity measures and directed connectivity analyses. Several studies used Shannon entropy to quantify decision uncertainty, choice difficulty, or behavioral inconsistency (Bernacer et al., 2019; Bobadilla-Suarez et al., 2020), while others employed sample entropy to assess signal complexity changes between rest and task states, particularly during working memory tasks (Nezafati et al., 2020). Transfer entropy was used in a subset of studies to examine directed information flow between brain regions during executive tasks, highlighting task-dependent changes in connectivity structure (Zuo et al., 2014). Overall, executive function studies demonstrated the broadest diversity of entropy implementations among all task contexts.

#### Learning and memory

Learning and memory tasks represented a moderate proportion of the included studies. Entropy-based analyses in this category were most commonly implemented using Shannon entropy, with additional use of sample entropy and multiscale entropy. In many studies, Shannon entropy was derived from computational or probabilistic models and used to quantify uncertainty, unpredictability, or information content during learning or decision formation (Davis et al., 2012). A smaller subset of studies employed entropy as a signal-complexity measure rather than a model-derived uncertainty metric. Multiscale entropy was used to assess changes in large-scale network complexity associated with encoding and post-encoding memory processes (McDonough et al., 2019), while sample entropy was applied to characterize differences in neural complexity related to developmental maturity during learning tasks (Amalric & Cantlon, 2023). Overall, studies in this category primarily used entropy to index learning-related uncertainty or information structure, with fewer studies focusing on changes in neural signal complexity across learning and memory states.

#### Language tasks

A limited number of studies examined language-related task contexts using entropy measures. All identified studies in this category employed Shannon entropy, typically within regional or whole-brain-level analytical scopes (Willems et al., 2016). Entropy was used to quantify variability associated with differences in task structure or stimulus predictability, although methodological approaches varied substantially.

#### Perceptual-motor control

Perceptual–motor control tasks represented the largest task-context category among the included studies. These studies employed a range of entropy measures, including Shannon entropy, sample entropy, approximate entropy, and transfer entropy. Several studies applied Shannon entropy directly to fMRI time series or activity distributions to quantify task-related changes in signal variability or information content during perceptual or motor engagement (De Araujo et al., 2003; Dinuzzo et al., 2017; Ponce-Alvarez et al., 2015). Other work employed Shannon entropy to characterize the information content or statistical structure of perceptual or auditory stimuli and related these measures to task-evoked brain responses (Alluri et al., 2012; Hoefle et al., 2018; Nastase et al., 2015; Tobia et al., 2012; Tremblay et al., 2013). In auditory–motor tasks, Shannon entropy was also used to quantify behavioral variability or entrainment during sensorimotor synchronization tasks (Siman-Tov et al., 2022). Approximate entropy was used to quantify temporal complexity of fine motor output across effectors (Holtrop et al., 2014), while Hilbert phase entropy was proposed as a phase-based activation detection method less sensitive to hemodynamic variability (Liao et al., 2010). Directed information-theoretic measures, including transfer entropy, were applied to reveal task-dependent changes in information flow within motor networks (Lizier et al., 2011). Other methodological work focused on entropy-based criteria for independent component estimation or multimodal fusion rather than task-related entropy effects per se (Li et al., 2021; Li et al., 2007; Li et al., 2006). Overall, perceptual–motor control studies exhibited the greatest methodological diversity in entropy implementation, spanning signal-level, distributional, behavioral, and connectivity-based approaches. This irregularity reflects both the prevalence of perceptual–motor tasks in task-based fMRI research and the flexibility of entropy-based methods for probing different aspects of task-related neural dynamics.

#### Social cognition

Studies investigating social cognition tasks applied either Shannon entropy or sample entropy. Entropy measures were used at regional or network levels to characterize variability associated with social or emotional task conditions (Takahashi et al., 2015). As with other categories, heterogeneity in task design and analytic implementation limited direct comparison across studies.

#### Multiple tasks

A subset of studies employed multiple task conditions, whole-brain task batteries, or combined task–rest designs that could not be clearly assigned to a single cognitive domain. These studies primarily used entropy-based or information-theoretic measures to characterize global, network-level, or system-wide brain dynamics, rather than task-specific cognitive operations (Deco et al., 2021; Gale et al., 2021; Omidvarnia et al., 2021).

Across this category, entropy measures were applied predominantly at the whole-brain, network, or graph-theoretic level, with a focus on quantifying signal complexity, information flow, or uncertainty across different task contexts. Several studies employed transfer entropy or related directed information-theoretic measures to examine task-dependent changes in bidirectional or causal information flow across large-scale networks, often across multiple tasks within the same dataset (Deco et al., 2021; Deleus et al., 2002). In these studies, entropy was used to identify integrative or coordinating network components that persisted across task conditions rather than to distinguish individual task effects.

Other studies applied Shannon entropy, sample entropy, multiscale entropy, or graph-based entropy measures to assess temporal complexity or network reconfiguration across task and rest states. These analyses commonly reported consistent spatial patterns of entropy or complexity that were modulated by task engagement, suggesting that entropy measures capture stable features of large-scale functional organization that generalize across task contexts (Gale et al., 2021; Omidvarnia et al., 2022; Sen et al., 2019).

Several studies further integrated entropy-based metrics within broader network models, including overlapping community detection, causal network inference, functional alignment, and communication-based network models. In these cases, entropy-related quantities were used to quantify uncertainty, diversity, or integration within complex network representations derived from multiple task conditions, rather than to interpret task-specific cognitive processes (Bazeille et al., 2021; Najafi et al., 2016; Seguin et al., 2022).

Overall, studies in this category used entropy measures as task-general descriptors of large-scale brain organization, emphasizing methodological characterization of neural complexity, information flow, and network integration across diverse task contexts. Task conditions were treated primarily as perturbations or contextual variations, with entropy-based analyses focusing on global dynamical properties rather than domain-specific cognitive interpretations.

Across all task contexts, Shannon entropy was the most consistently applied measure, whereas other entropy types were used more selectively depending on analytic scope and methodological intent. The distribution of entropy measures across task contexts highlights substantial discrepancy in implementation, reinforcing that task categorization serves a descriptive role rather than a basis for theoretical inference. These observations further motivate the methodological synthesis presented in subsequent sections.

### Parameterization practices

Across the 92 included studies, parameterization practices varied substantially both within and between entropy families, and reporting ranged from highly explicit (e.g., listing all hyperparameters and estimation settings) to minimal (e.g., naming the entropy type without specifying implementation details). These differences are most consequential for measures that require user-defined parameters, particularly sample entropy (SampEn), approximate entropy (ApEn), multiscale entropy (MSE), and directed information-flow measures such as transfer entropy (TE) and normalized directed transfer entropy (NDTE), because reasonable but different parameter choices can shift absolute entropy values and sometimes even alter contrasts across task conditions.

For SampEn and ApEn analyses, the two principal tunable parameters, the embedding dimension (m) and the tolerance threshold (r), were inconsistently selected across studies. Several task-based fMRI studies adopted conventional parameterizations such as m = 2 with r set to a fraction of the signal standard deviation, often *r* = 0.2 or *r* = 0.5 × SD (Sokunbi et al., 2011; Wang et al. 2014b). Other studies employed higher embedding dimensions and larger tolerances, such as m = 3 and *r* = 0.6 × SD, motivated by efforts to improve consistency under short time-series constraints (Nezafati et al., 2020). Developmental task-based fMRI studies further illustrated how data-length limitations influenced parameter selection. Amalric and Cantlon (2023) explicitly used m = 2 and *r* = 0.5 × SD, noting that shorter task runs restrict the reliable estimation of higher-dimensional templates. Their parameter choices were grounded in prior recommendations suggesting that for m = 2, tolerance values in the approximate range of 0.25–0.65 × SD provide a reasonable balance between sensitivity and statistical stability. A smaller subset of studies treated parameter selection as an empirical problem rather than a fixed convention. For example, Wang et al. (2014a) systematically evaluated multiple combinations of m and r and demonstrated that entropy estimates and their discriminative power across task conditions were sensitive to these choices. Despite this, formal sensitivity analyses or reliability checks across parameter grids were uncommon in the broader literature. Moreover, it is worth noting that outside the fMRI literature, particularly in physiological and EEG applications, commonly applied tolerance thresholds for SampEn and ApEn are often lower, typically in the range of approximately 0.1–0.25 × SD (Burioka et al., 2005; Ni et al., 2013; Zhou et al., 2012). These differences likely reflect fundamental disparities in signal properties because high–temporal-resolution physiological signals such as EEG typically yield long time series whereas the shorter temporally smoothed and autocorrelated nature of fMRI BOLD data often requires larger tolerance values to achieve sufficient template matches and stable entropy estimation (Sokunbi, 2014).

Multiscale entropy analyses showed more inconsistency in parameters beyond those inherent to SampEn. Studies differed in the selection of scale factors (τ), the coarse-graining procedure used to generate scale-specific time series, and the method by which multiscale entropy curves were summarized for statistical analysis. Some studies explicitly specified parameters such as m = 2, *r* = 0.5, and τ_max = 10, and summarized complexity using an area-under-the-curve “complexity index” normalized by the maximum scale (McDonough et al., 2019). In contrast, other studies constrained their analyses to lower scales due to limited time-series length, noting that reliable entropy estimation at higher scales was not feasible for typical task-based fMRI acquisitions (Amalric & Cantlon, 2023). These differences are methodologically meaningful, as broader scale ranges emphasize slower temporal dynamics, whereas scale-restricted analyses predominantly capture local signal irregularity. However, explicit justification for scale selection and curve summarization strategies was inconsistently reported across MSE studies. Studies employing transfer entropy or related directed information metrics introduced further parameterization complexity, including estimator choice such as discrete versus continuous, embedding or history length, temporal lag, and statistical inference procedures. Several NDTE-based frameworks explicitly reported their lag parameters and surrogate-testing strategies, often using fixed lags (lag = 10) and large numbers of surrogate time series to establish significance thresholds (Deco et al., 2021). Other TE studies relied on continuous-variable estimators, such as k-nearest-neighbor or kernel-based approaches, with estimator-specific parameters (neighborhood size or window width) influencing information-flow estimates (Frässle et al., 2017; Lizier et al., 2011). Although surrogate-based validation was frequently described as essential for inference, the number of surrogates, construction methods, and correction strategies varied substantially and were not uniformly reported. This inconsistency limits direct comparison across directed-connectivity studies, particularly between pairwise and multivariate implementations.

While Shannon entropy is often perceived as comparatively parameter-light, substantial analytic flexibility remained in how probability distributions were estimated from continuous fMRI data. Several studies explicitly employed kernel density or Parzen window estimation to derive probability density functions prior to entropy computation (De Araujo et al., 2003; Dinuzzo et al., 2017). Others relied on implicit binning strategies or entropy estimates derived from model-based regressors representing stimulus or decision uncertainty (Davis et al., 2012; Willems et al., 2016). These choices such as bin width, kernel bandwidth, normalization, and whether entropy was computed on raw BOLD amplitudes, residuals, or derived features function as hidden parameters that can substantially affect entropy magnitude and interpretability. Nevertheless, such details were often omitted or only briefly described, despite their relevance for reproducibility.

Collectively, the reviewed literature demonstrates that entropy parameterization practices in task-based fMRI research remain largely study-specific rather than standardized. The strongest methodological practices observed included complete disclosure of entropy definitions, explicit reporting of all hyperparameters (e.g., m, r, τ_max, lag), justification based on data-length constraints, and validation through sensitivity analyses or surrogate testing (Amalric & Cantlon, 2023; Deco et al., 2021; Wang et al. 2014b). In contrast, incomplete parameter reporting and the absence of reliability checks were common and represent a primary barrier to cross-study comparability. These findings underscore the need for standardized reporting conventions and routine sensitivity analyses when applying entropy measures to task-based fMRI data, particularly for methods with multiple free parameters.

### Risk of bias assessment

Across the 92 included task-based fMRI entropy studies, risk-of-bias considerations were dominated by motion and preprocessing decisions that directly alter BOLD temporal structure, analytic flexibility in entropy parameterization and temporal sampling, and multiplicity and statistical thresholding choices in voxel-wise and network-wide inference. Overall, most studies used conventional fMRI acquisition and preprocessing pipelines, but reporting depth and the degree of validation against null/surrogate expectations varied substantially across the literature (Bainbridge & Oliva, 2015; De Araujo et al., 2003; Fuhrmann Alpert et al., 2007; Li et al., 2007; Li et al., 2006; Nastase et al., 2015; Omidvarnia et al., 2022; Ponce-Alvarez et al., 2015; Sturzbecher et al., 2009; Tobia et al., 2012; Tremblay et al., 2013).

#### Preprocessing

Because entropy metrics are sensitive to temporal regularity and variance structure, preprocessing constitutes a major potential bias pathway. Many studies applied standard steps such as slice-timing correction, motion correction, temporal filtering, and spatial smoothing, which can each change the autocorrelation and amplitude distribution of BOLD time series. For instance, a typical pipeline included trilinear motion correction, temporal high-pass filtering, and spatial smoothing (Bainbridge & Oliva, 2015; De Araujo et al., 2003; Dinuzzo et al., 2017; Holtrop et al., 2014). More aggressive denoising approach such as ICA-based removal of motion-related components were also present in the corpus and can reduce motion artifacts but may also remove neural signal of interest if not carefully validated. Examples include ICA-driven or component-based preprocessing pipelines described in methodological entropy and multimodal fusion studies (Li et al., 2021; Y. O. Li et al., 2007; Y.-O. Li et al., 2006; Omidvarnia et al., 2022).

A related source of inconsistency is also found in parcellation and signal aggregation, which changes the effective signal-to-noise ratio and can inflate comparability challenges across studies. For example, cortical or network-level aggregation prior to entropy estimation was explicitly adopted in whole-brain or network-complexity analyses (Gale et al., 2021; Omidvarnia et al., 2022; Sen et al., 2019), whereas other studies relied on voxelwise entropy or MI mapping (Fuhrmann Alpert et al., 2007; Sturzbecher et al., 2009), producing inherently different sensitivity and bias profiles.

#### Temporal sampling and data-length

Entropy estimation reliability depends strongly on time-series length and TR. In task-based fMRI, short runs or conditions with limited volumes can produce unstable or biased entropy estimates, especially for multiscale approaches and directed information-flow measures. Several studies explicitly noted constraints imposed by task duration or temporal resolution when selecting entropy formulations or scale ranges (Fuhrmann Alpert et al., 2007; Gale et al., 2021; Holtrop et al., 2014; McDonough et al., 2019; Omidvarnia et al., 2022). Differences in run duration, number of trials, and condition segmentation introduce unequal effective sample sizes across tasks and conditions, raising risks of biased cross-condition or cross-study comparisons unless time-series length is matched or explicitly controlled (Nastase et al., 2015; Tobia et al., 2012; Tremblay et al., 2013).

#### Parameter

A central risk-of-bias issue was inconsistent parameter reporting and flexible analytic decisions (e.g., embedding choices, discretization/binning strategies, scale settings, and window definitions). In mutual-information–based event-related mapping, bin number, window width, and latency search ranges were tuned and then used for voxelwise inference (Deleus et al., 2002; Fuhrmann Alpert et al., 2007). Non-extensive entropy approaches introduced additional free parameters (the Tsallis q parameter and discretization levels), with parameter optimization often based on simulations or empirical heuristics (Sturzbecher et al., 2009). While such tuning can be principled, it also creates researcher degrees of freedom. The risk is lowest when choices are justified theoretically, validated on held-out or surrogate data, and fully reported (Li et al., 2007; Li et al., 2006; Ponce-Alvarez et al., 2015).

#### Assumption bias

Several information-theoretic methods were explicitly motivated as alternatives to GLM-style approaches that assume a canonical HRF and linear stimulus–BOLD mapping. For example, entropy and mutual information-based fMRI methods were presented as reducing sensitivity to HRF-shape misspecification and latency variability (Fuhrmann Alpert et al., 2007; Li et al., 2006; Sturzbecher et al., 2009). However, these approaches still inherently contain assumptions (e.g., discretization schemes, window placement, estimator choice), so the bias tradeoff shifts from HRF-model misspecification toward parameter and estimator sensitivity (De Araujo et al., 2003; Dinuzzo et al., 2017; Li et al., 2007).

#### Statistical Inference, Multiple Comparisons, and Thresholding

Voxel-wise entropy requires strict control of false positives. Some approaches estimated thresholds using permutation or randomization procedures tied to the dataset, improving calibration but depending strongly on null construction (Fuhrmann Alpert et al., 2007; Ponce-Alvarez et al., 2015; Sturzbecher et al., 2009). Elsewhere, ROI-based aggregation or relaxed thresholds were adopted when strict voxel-wise correction yielded null results, which can introduce selective reporting risk if not transparently documented (Bainbridge & Oliva, 2015; Tobia et al., 2012; Tremblay et al., 2013). Variability in multiple-comparison correction strategies was evident across perceptual, motor, auditory, and executive task studies (Hoefle et al., 2018; Nastase et al., 2015; Siman-Tov et al., 2022).

#### Bias of validation against null/surrogate expectations

Only a subset of studies developed null distributions or surrogate data explicit as part of entropy or complexity analysis. Surrogate-based validation was highlighted as critical in distinguishing meaningful neural complexity from stochastic variability in several methodological and large-scale network studies (Deco et al., 2021; Gale et al., 2021; Lizier et al., 2011; Omidvarnia et al., 2021). In contrast, many task-based applications relied primarily on conventional parametric inference without explicit surrogate testing, contributing to inconsistency in logical soundness (De Araujo et al., 2003; Tobia et al., 2012; Tremblay et al., 2013).

In sum, the principal methodological risks were not tied to any single cognitive domain, but to a recurring pattern. Those include preprocessing and denoising choices that reshape BOLD temporal structure (Bainbridge & Oliva, 2015; Dinuzzo et al., 2017; Holtrop et al., 2014), data-length limitations that constrain reliable entropy estimation (Fuhrmann Alpert et al., 2007; Gale et al., 2021; McDonough et al., 2019), and analytic flexibility in entropy or MI parameterization and statistical thresholding (Y. O. Li et al., 2007; Li et al., 2006; Ponce-Alvarez et al., 2015; Sturzbecher et al., 2009). Consequently, narrative synthesis across studies is most defensible when emphasizing within-study contrasts rather than absolute entropy magnitudes, and when prioritizing findings supported by transparent parameter reporting and, where feasible, permutation or surrogate-based validation (Deco et al., 2021; Fuhrmann Alpert et al., 2007; Lizier et al., 2011; Omidvarnia et al., 2022).

### Certainty of evidence

The overall certainty of evidence across the included studies was assessed qualitatively, taking into account study design, consistency of findings, methodological variations, and identified sources of potential bias. Given the diversity of entropy measures, task conditions, and analytical approaches, the certainty of evidence was generally judged to be moderate (e.g., (Dinuzzo et al., 2017; Lizier et al., 2011; Tobia et al., 2012)). Most included studies were exploratory in nature and employed cross-sectional designs, limiting causal inference (e.g., (Lizier et al., 2011; Tobia et al., 2012; Zhang & Rowe, 2015). While many studies reported statistically significant entropy-related effects within specific task contexts, consistency across studies was difficult to establish due to substantial disparities in entropy implementation, parameterization, and outcome measures (Dinuzzo et al., 2017; Lizier et al., 2011; Rodriguez et al., 2015; Tobia et al., 2012). As a result, direct replication of findings across independent datasets was uncommon (Mack et al., 2020; Zhang & Rowe, 2015). Confidence in the evidence was further influenced by variability in reporting practices and limited use of reliability checks or sensitivity analyses. Although several studies incorporated validation procedures such as permutation testing, surrogate-data testing, or simulation-based analyses, these practices were not uniformly applied (Lizier et al., 2011; Zhang & Rowe, 2015). Differences in sample size and time-series length also contributed to uncertainty in the stability of entropy estimates, particularly for approaches requiring reliable estimation over many comparisons or multiple scales (Lizier et al., 2011; Zhang & Rowe, 2015). Despite these limitations, the evidence base demonstrated convergent support for the feasibility and flexibility of entropy-based approaches in task-based fMRI research. Across multiple cognitive domains, entropy measures were successfully applied to characterize task-related variability, uncertainty, and complexity, suggesting that entropy-based methods provide a viable analytical scopes when appropriately implemented and reported (Dinuzzo et al., 2017; Lizier et al., 2011; Tobia et al., 2012). Overall, while the certainty of evidence supporting specific entropy–task relationships remains limited by methodological heterogeneity, the collective findings support the continued use of entropy-based analyses in task-based fMRI, with the expectation that future studies employing standardized reporting and validation practices will strengthen the evidence base (Lizier et al., 2011; Mack et al., 2020; Rodriguez et al., 2015).

## Discussion

This systematic review synthesized 92 task-based fMRI studies applying entropy-based measures to characterize neural signal complexity, variability, and information flow in healthy human participants. Rather than adjudicating domain-specific neurocognitive claims, the present discussion interprets the findings in methodological terms, focusing on how entropy measures are selected, parameterized, and analytically deployed in task-based fMRI research. Viewed through this lens, the literature demonstrates both the versatility of entropy-based approaches and substantial barriers to cross-study comparability and reproducibility.

Overall, it is essential to clarify that “entropy” does not represent a single, uniform construct across task-based fMRI studies. Instead, different entropy formulations operationalize distinct methodological and conceptual quantities, including temporal signal regularity (approximate and sample entropy), multi–time scale complexity (multiscale entropy), distributional uncertainty or information content (Shannon entropy), and directed information flow between brain regions (transfer entropy). These measures are therefore not interchangeable, and differences in their mathematical definitions and estimation procedures imply fundamentally different interpretations of what is being quantified. Much of the heterogeneity observed across studies reflects these divergent meanings rather than inconsistency in empirical findings. Recognizing this distinction is critical for understanding why entropy measures are applied at different analytical scopes and with different parameterization practices across the literature. This framework provides context for the methodological patterns and limitations discussed below.

Consistent with the distinction outlined above, a central finding of this review is that entropy measures are not used interchangeably across studies but instead occupy distinct methodological roles. Shannon entropy dominates the task-based fMRI literature due to its conceptual simplicity, analytical flexibility, and compatibility with probabilistic models. It has been applied to quantify signal variability, stimulus uncertainty, decision entropy, and information content derived from computational models, and it is frequently embedded within machine-learning or classification pipelines. However, when computed on marginal amplitude distributions or static probability estimates, it abstracts away temporal dependencies in the BOLD signal. Importantly, this limitation is methodological rather than inherent to Shannon entropy itself. Several studies compute entropy over state sequences, sliding temporal windows, or within Markov and state-space frameworks (Vidaurre et al., 2017; Xin et al., 2024), thereby partially reintroducing temporal structure and capturing time-dependent uncertainty. These sequence-based implementations increase sensitivity to temporal dynamics but also introduce additional modeling assumptions and parameter choices. Consequently, the interpretability of Shannon entropy in task-based fMRI depends critically on how probability distributions are constructed and whether temporal information is explicitly incorporated. Future studies would benefit from explicitly stating whether Shannon entropy is computed on marginal distributions or temporal sequences, as these choices imply fundamentally different interpretations of neural variability.

In contrast, approximate entropy and sample entropy are primarily used to quantify local temporal regularity in BOLD time series. Their methodological strength lies in capturing short-range predictability and irregularity, but this strength is coupled with sensitivity to parameter selection and time-series length (Sokunbi, 2014). Multiscale entropy extends this by probing complexity across multiple temporal scales, offering a richer description of hierarchical neural dynamics but at the cost of increased data-length requirements and additional analytic decisions (Amalric & Cantlon, 2023). Transfer entropy and related directed information-theoretic measures serve a distinct purpose altogether, enabling estimation of directed information flow and task-dependent changes in effective connectivity. These measures are analytically powerful but introduce further complexity through estimator choice, embedding parameters, temporal lags, and surrogate-based inference. Collectively, these findings reinforce that entropy measures should be understood as methodological instruments tailored to specific analytic goals, rather than as generic markers of “brain complexity.” Failure to articulate this distinction contributes to conceptual ambiguity and limits the interpretability of entropy findings across studies.

One of the most consistent patterns across the reviewed literature is the extensive differences in practices of choosing parameters, particularly for entropy measures requiring user-defined hyperparameters. For sample entropy and approximate entropy, embedding dimension (m) and tolerance (r) varied widely across studies, often without explicit justification or sensitivity analysis. While several studies converged on commonly recommended values (e.g., m = 2–3 and *r* ≈ 0.5–0.6 × SD), others demonstrated that reasonable alternative choices could meaningfully alter entropy estimates and task contrasts. Despite this, systematic exploration of parameter sensitivity remained the exception rather than the norm. Multiscale entropy analyses exhibited even greater variability, with differences in scale range, coarse-graining procedures, and summary metrics (e.g., complexity index versus scale-specific comparisons) (Amalric & Cantlon, 2023). Importantly, these choices determine whether MSE primarily captures local irregularity or slower, large-scale temporal structure. Directed entropy measures introduced additional layers of analytic flexibility, including estimator type, lag selection, embedding history, and surrogate-testing procedures. Although several studies emphasized surrogate-based validation as essential for inference (Deco et al., 2021), reporting practices varied widely, limiting reproducibility. Even Shannon entropy, often perceived as parameter-light, involved substantial implicit analytic choices such as binning strategies, kernel density estimation, normalization procedures, and decisions about whether entropy was computed on raw BOLD signals, residuals, or model-derived regressors (Davis et al., 2012; De Araujo et al., 2003; Dinuzzo et al., 2017; Willems et al., 2016). These choices function as hidden parameters that can substantially influence results but were frequently underreported. Taken together, parameterization inconsistency emerges as the dominant barrier to cross-study comparability. This finding aligns with recent methodological critiques emphasizing that differences in entropy definitions, estimation procedures, and preprocessing pipelines can overshadow substantive neural effects if not explicitly controlled or reported.

Moreover, entropy measures are inherently sensitive to the temporal and statistical structure of input signals, making preprocessing choices a critical source of bias. Motion correction, temporal filtering, spatial smoothing, and denoising approaches can all alter autocorrelation structure and variance, directly impacting entropy estimates. While most studies employed standard fMRI preprocessing pipelines, the depth of reporting and validation varied substantially (Fuhrmann Alpert et al., 2007; Gale et al., 2021; Holtrop et al., 2014; McDonough et al., 2019; Omidvarnia et al., 2022). More aggressive denoising strategies, such as ICA-based component removal, may reduce motion-related artifacts but also risk removing neural signal relevant to entropy estimation if not carefully evaluated. Temporal sampling constraints further complicate entropy estimation in task-based fMRI. Short task blocks, limited run durations, and relatively long TRs can bias entropy estimates, particularly for multiscale and directed measures. Several studies explicitly acknowledged these constraints and adjusted their analytic choices accordingly, but such considerations were not uniformly addressed across the literature. These factors underscore that entropy measures cannot be meaningfully interpreted without reference to data length, sampling rate, and preprocessing context.

The findings of this review suggest that the field would benefit more from methodological standardization and transparency than from further proliferation of entropy applications across increasingly diverse tasks. At minimum, future task-based fMRI studies employing entropy measures should explicitly report:

1. Full mathematical definition of the entropy measure used.
2. All parameter values and estimation settings.
3. Justification for parameter choices in relation to data length and task design.
4. Reliability checks such as sensitivity analyses or surrogate testing where appropriate.

Without these elements, entropy findings remain difficult to interpret, replicate, or synthesize. Importantly, this review also highlights that organizing entropy findings by cognitive domain, while descriptively convenient, risks obscuring the methodological focus of entropy-based research. Task conditions should be treated primarily as experimental contexts in which analytic methods are deployed, rather than as the primary axis for theoretical interpretation. This perspective aligns more closely with the technical objectives of methodological reviews and avoids overextension into domain-specific neurocognitive claims that are better addressed by targeted meta-analyses.

Several limitations should be acknowledged. First, this review synthesized a methodologically diverse literature, precluding quantitative meta-analysis or formal effect-size comparisons. Second, despite efforts to focus on technical implementation, the available studies often conflated entropy as a signal-complexity metric with entropy as a model-derived uncertainty measure, complicating synthesis. Third, although risk of bias was assessed qualitatively, the absence of standardized entropy reporting limited the precision of bias judgments. Finally, while PRISMA 2020 guidelines were followed, the review was not preregistered, and no automation tools were used in screening or data extraction.

## Conclusion

Entropy-based analysis of fMRI data has become an increasingly influential approach for examining brain function, providing a refined perspective on neural complexity in healthy and clinical populations. This review underscores the versatility of entropy measures, illustrating their utility in characterizing cognitive processing, functional connectivity, and pathology-related alterations. The evidence supports the view that entropy extends beyond a simple index of randomness, instead reflecting the brain’s capacity for dynamic adaptation to cognitive demands and disease states. In healthy cohorts, entropy metrics yield important insights into cognitive efficiency, adaptability, and domain-specific functional specialization, with entropy fluctuations aligning with established neural networks.

Despite these advances, notable challenges remain in incorporating entropy into mainstream neuroimaging research. Differences in entropy definitions, computational methodologies, and experimental designs currently hinder cross-study comparability and the development of standardized entropy biomarkers (Ray et al., 2023).

Taken together, entropy analysis represents a promising direction in neuroscience, linking neural complexity with cognitive function. With ongoing methodological improvements and interdisciplinary collaboration, entropy metrics have the potential to deepen our understanding of brain organization and to inform precision diagnostics and targeted therapeutic strategies for neurological and psychiatric disorders (Huang et al., 2024).

## Future work

Future investigations will be directed toward addressing these limitations. A more granular evaluation of specific cognitive domains will be undertaken to delineate the distinct contributions of various entropy measures to different facets of brain function. To enhance the representativeness of the analysis, the review will extend beyond the most frequently cited works to encompass the full range of available studies within each category. Furthermore, empirical validation will be pursued using data from the Human Connectome Project (HCP). By systematically applying the five principal entropy measures to this dataset, we aim to assess their respective capacities to characterize neural dynamics and connectivity patterns across diverse contexts. This approach is expected to reinforce the consistency of entropy-based metrics and facilitate the establishment of standardized methodological guidelines for their implementation in future neuroimaging research.

## Data Availability

No datasets were generated or analysed during the current study.
